# Manipulation of Glucose Availability to Boost Cancer Immunotherapies

**DOI:** 10.3390/cancers12102940

**Published:** 2020-10-12

**Authors:** Federica Marchesi, Debora Vignali, Beatrice Manini, Alessandra Rigamonti, Paolo Monti

**Affiliations:** 1 Center-IRCCS, Department of Immunology and Inflammation, Humanitas Clinical and Research, Rozzano, 20089 Milan, Italy; Federica.Marchesi@humanitasresearch.it (F.M.); alessandra.rigamonti@humanitasresearch.it (A.R.); 2 Department of Medical Biotechnology and Translational Medicine, University of Milan, 20133 Milan, Italy; 3 San Raffaele Diabetes Research Institute, IRCCS Ospedale San Raffaele, 20131 Milan, Italy; vignali.debora@hsr.it (D.V.); b.manini@studenti.unisr.it (B.M.); 4 San Raffaele Vita Salute University, 20133 Milan, Italy

**Keywords:** immune therapies, glucose metabolism, T cells, Glut1

## Abstract

**Simple Summary:**

Substantial effort has been made in recent years to improve the clinical outcome of cancer immunotherapy. Recent findings suggest that manipulation of glucose metabolism can represent a valuable tool to limit cancer cell growth and to help the immune system to elicit an efficient and protective response to cancer cells. Both pharmacological approaches and diets with a low carbohydrate content are under evaluation in order to limit glucose availability in metabolic processes for a future application as co-adjuvant strategies to improve cancer immunotherapies.

**Abstract:**

The orchestration of T cell responses is intimately linked to the execution of metabolic processes, both in homeostasis and disease. In cancer tissues, metabolic alterations that characterize malignant transformation profoundly affect the composition of the immune microenvironment and the accomplishment of an effective anti-tumor response. The growing understanding of the metabolic regulation of immune cell function has shed light on the possibility to manipulate metabolic pathways as a strategy to improve T cell function in cancer. Among others, glucose metabolism through the glycolytic pathway is central in shaping T cell responses and emerges as an ideal target to improve cancer immunotherapy. However, metabolic manipulation requires a deep level of control over side-effects and development of biomarkers of response. Here, we summarize the metabolic control of T cell function and focus on the implications of metabolic manipulation for the design of immunotherapeutic strategies. Integrating our understanding of T cell function and metabolism will hopefully foster the forthcoming development of more effective immunotherapeutic strategies.

## 1. Introduction

Targeting metabolic pathways is emerging as a potent strategy to manipulate immune responses against cancer [1]. The mechanistic explanation behind this approach is provided by the fact that immune cell activation, differentiation, and function necessitate unique metabolic requirements to support both the energetic and biosynthetic demands. Adoptively transferred T cells are a potent therapeutic tool for the eradication of established tumors and provide long-term immunity, protecting the individual from disease recurrence [2]. Importantly, both the effector function and generation of memory responses are intimately linked to specific metabolic processes [3], suggesting that the metabolic status of transferred T cells is a critical factor to achieve clinical response. While the differentiation of effector T cells and their capacity to effectively eliminate target cells are related to glycolysis, the suppression of glycolysis is involved in the generation and persistence of memory T cells, which rely on oxidative phosphorylation [4]. Glucose metabolism through the glycolytic pathway is therefore central in shaping T cell responses and is therefore an ideal target to improve cancer immunotherapy. 

On the other hand, tumor cells are often dependent on glucose as a primary energy source, due to their extensive proliferation that necessitates uninterrupted access to energy and the building blocks of cellular biomass. To meet these requirements, cancer cells utilize glycolysis, even in the presence of oxygen, a process referred to as aerobic glycolysis or the “Warburg effect”. Collectively, targeting glucose metabolism also has a potential benefit in controlling tumor growth and spreading [5,6].

An additional advantage of targeting glucose metabolism is the availability of a broad arsenal of molecules and drugs. Several inhibitors of glycolysis have been developed over the years, including 2-deoxiglucose. More recently, a novel class of small molecules displaying high selectivity against glucose transporter 1 (Glut1) and with good pharmacokinetic and pharmacodynamic characteristics have been produced [7]. The pharmacological blockade of Glut1 is therefore a promising strategy to boost both a long-lasting immune response and reduce tumor growth. In addition to pharmacological targeting, glucose metabolism can also be controlled through the diet. Low-carb and ketogenic diets have been proposed as adjuvants to standard anticancer treatments such as chemotherapy and radiotherapy [8]. The hypothesis is that a reduced intake of carbohydrates can limit the availability of glucose for tumor growth and, despite the fact that clinical data is still controversial, there is a considerable effort in this field.

As we will discuss throughout this review, targeting glucose metabolism concomitantly provides an opportunity to improve the longevity of the anti-tumor T cell response and to contrast tumor growth, thus representing a therapeutic option to be contemplated in immunotherapeutic strategies. Nonetheless, considering that T cells rely on glucose metabolism for their activation, glucose-modulating therapies may concomitantly support and hamper anti-tumor immunity [9], suggesting that predictive biomarker-based approaches should be implemented. Moreover, potential side effects, off-target effects, and the complexity of the whole-body metabolism can interfere with the effectiveness of a metabolic manipulation in cancer settings. Collectively, metabolic targeting is not meant to affect a specific cell but rather the metabolic processes that sustain disease progression.

## 2. Fundamentals of Cancer Metabolism

In order to achieve and sustain their proliferative capacity, cancer cells must enhance metabolic pathways, using available nutrients to sustain energy demand, redox balance, and biosynthesis. Glucose is a primary source of energy and biosynthesis intermediates for all cells. Normal cells typically convert glucose into pyruvate that is subsequently transported into the mitochondria to enter the tricarboxylic acid (TCA) cycle, with a high-energy yield in the form of adenosine triphosphate (ATP). In contrast, cancer cells convert much of the pyruvate into lactate but with a very low energy yield. This difference accounts for their high glucose consumption rate, which is needed to fulfill their metabolic demand. A high rate of glucose catabolism into lactate is a metabolic hallmark of cancer cells and has been observed in the majority of cancers, as first reported by Warburg [10]. However, several other metabolic pathways are active in cancer cells and are of fundamental importance in cancer cell growth and as potential targets for therapeutic intervention. The catabolism of aminoacids is an important metabolic pathway often de-regulated in cancer cells. Glutamine is the most abundant circulating amino acid in humans and the second most consumed nutrient—after glucose—by cancer cells in vitro [11]. Glutamine can be hydrolyzed to glutamate and inorganic ammonia by the action of glutaminases and further converted by glutamate dehydrogenase or transaminases into α-ketoglutarate, a substrate of the TCA cycle. The inhibition of glutaminases has shown the inhibition of cancer cell growth in several cancer models, and one such inhibitor is currently under investigation in clinical trials [12]. However, the pro-growth effect of glutamine is not solely explained by carbon metabolism, and other effects of glutamine in nitrogen metabolism and signaling events are currently under investigation to understand the complex link between glutamine and cancer cell growth [13]. Other amino acids and related metabolic pathways have been found to contribute to cancer cell growth, including serine, methionine, arginine, and ornithine, whose contribution to tumor metabolism has been reviewed elsewhere [14]. Although most cancer cells display a high glycolytic rate, they still retain also a mitochondrial oxidative phosphorylation activity [15]. Despite the fact that this activity requires oxygen, which is often limited in tumor tissues, nutrients such as glucose and glutamine are commonly used in oxidative metabolic pathways. Mitochondrial metabolism is also under investigation as a potential therapeutic target for the inhibition of cancer cell growth. As an example, metformin, the electron transport chain complex I inhibitor, is currently under investigation for cancer treatment [16]. The production of reactive oxygen species (ROS) is another important aspect of mitochondrial metabolism in cancer. At high concentrations, ROS can damage several cell components and normal cells have developed pathways to scavenge ROS. Glutathione is a major pathway for ROS detoxification in cancer cells, and the targeting of the ROS scavenging machinery is considered a potential target for therapies that aim at inhibiting cancer growth [17]. While several metabolic pathways are active in cancer cells and represent promising targets for metabolic intervention, the focus of this review is glucose metabolism with the possibility that targeting multiple metabolic ways could result in additive or synergistic effects in the impairment of cancer cell growth.

## 3. Basics of T Cell Metabolism

The T cell compartment is composed of several subsets, characterized by different phenotypes, functions, and a differentiation stage. Conventionally, T cells are divided into naïve T cells (Tn), central memory (Tcm), effector memory (Tem), or terminally differentiated effector memory (Temra), according to the expression of a core-set of surface markers, including CCR7, CD45RA, and CD62L [18]. More recently, the existence of a memory precursor with stem cell like properties (stem memory T cells, Tscm) has been documented in mice [19], non-human primates [20], and humans [21]. T cell subsets circulate in the blood, lymphoid organs, and tissues as resting cells, with a distinct homing capacity and life span. All memory subsets differentiate from a Tn precursor according to a progressive differentiation model [22]. In the steady state, T cells are quiescent and have low energetic and biosynthetic demands to fulfill housekeeping functions and the homeostatic turnover of macromolecules. Survival, homeostasis, and bio-energetic metabolism of quiescent T cells is primarily regulated by the availability of the homeostatic cytokine interleukin-7 (IL-7) [23], which promotes glucose uptake and use by favoring Glut1 trafficking [24] and acting as a transcriptional regulator of the hexokinase II genes [25]. IL-7 is required to sustain the basal metabolism in Tn and the absence of IL-7 signaling results in reduced metabolic activity and cell atrophy [26]. Another metabolic feature of memory T cells is the greater mitochondrial mass and consequent enhanced spare respiratory capacity (SRC) as compared to Tn [27]. Indeed, a hallmark of memory T cells is the capacity to develop a rapid recall response to antigens, which needs an immediate metabolic support provided by the mitochondrial cell mass. Moreover, memory T cells use glucose or glycerol for lipid synthesis that can be used to fuel fatty acid oxidation (FAO) and provide intermediate metabolites for the tricarboxylic acid cycle (TCA). Glycerol is internalized from the intracellular milieu using the glycerol channel aquaporin 9 (AQP9), while glucose uptake is mediated by the glucose transporter Glut1; the surface expression of both molecules depends on IL-7 signaling [28]. Different metabolic profiles of naïve and memory T cell subsets also reflect their differences in homeostatic regulation, life-span, and turnover in vivo. While naïve T cells have a half-life of approximately two years and do not proliferate, memory T cells have a half-life of approximately three weeks [29]. The metabolic signature of quiescent naïve and memory T cells largely relies on oxidative phosphorylation even though glucose is essential to maintain T cell homeostasis.

The recognition of peptide antigen/MHC complex by the T cell receptor (TCR), together with appropriate co-stimulation and cytokine signals, activates quiescent naïve and memory T cells. This activation process entails the triggering of intense proliferation, generating a large amount of effector T cells (Teff) from a single clone and the execution of effector functions, including the capacity to eliminate target cells and the production of B cell activating cytokines. The rate of T cell proliferation during the clonal expansion phase is unique in the body and requires an adequate amount of energy and bio-synthetic intermediates, these are achieved by the suppression of oxidative phosphorylation (OXPHOS) and the activation of glycolysis [30]. This metabolic switch involves the overexpression of the glucose transporter Glut1 to incorporate a larger amount of glucose [31] and the production of lactate, indicating that the pyruvate generated during glycolysis is not transported to the mitochondria for further oxidation [32]. 

Metabolic adaptations do not only regulate T cell proliferation during effector T cell responses but also control the generation of different T cell subsets. When Tn are activated by the recognition of the cognate antigen, a glycolytic metabolism favors the generation of a full effector progeny, whereas an oxidative metabolism or an inhibition of glycolysis induces the generation of memory T cells. The glucose analog 2-(N-[7-nitrobenz-2-oxa-1, 3-diazol-4-yl]amino)-2-deoxyglucose (2-NBDG) was used to sort highly glycolytic (2-NBDG^hi^) and poorly glycolytic (2-NBDG^lo^) p-mel-1 splenocytes [33]. Once adoptively transferred into wild type mice immunized with GP100, 2-NBDG^low^ exhibited poor engraftment, survival, and proliferation capacities, whereas 2-NBDG^lo^ cells exhibited 10- to 100-fold higher frequency over the time course of infection. The manipulation of the glycolytic metabolism heavily affects effector functions and memory formation in CD8+ T cells. CD8+ T cells transduced with the glycolytic enzyme Pgam1, which enhances glycolytic flux, acquire a full effector phenotype but have a decreased long-term survival in vivo [33]. On the other hand, blocking glycolysis with 2DG enforces a transcriptional program characteristic of memory cells, associated with enhanced engraftment and expansion when adoptively transferred. Mitochondrial dynamics can also influence the differentiation of T cells. Buck M. et al. showed that by altering cristae morphology, fusion events in memory T cells lead to the association of the electron transport chain complexes (ETC), favoring OXPHOS and FAO, while fission events in Teff were correlated with cristae expansion, a reduced ETC efficiency and a promoted glycolysis activity [34]. Taken together, these data suggest the possibility to manipulate the course of the T response, favoring a fully effector instead of a memory T cell response. Manipulation of glucose metabolism appears to be particularly effective to modify the fate of activated T cells.

## 4. Metabolism of T Regulatory Cells

Regulatory T cells (Treg) are a sub-population of T cells with a fundamental role in immune regulation and homeostasis. Treg are characterized by the expression of the co-receptor CD4, the cytokine receptor CD25, and the transcription factor Foxp3, which is a key element of Treg differentiation and their suppressive function. The suppressive capacity of Treg is commonly associated with the amount of FOXP3 expression. Treg can play an important role in cancer, as they abundantly infiltrate tumor tissues, and their density is commonly associated with poor prognosis [35], with the important exception of human colo-rectal cancer [36,37]. The removal of Treg enhances anti-tumor immune responses and they can be specifically targeted for augmenting tumor immunity [38]. Metabolic differences between Treg and conventional T cells have been investigated, suggesting the possibility to differentially affect the two subsets to enhance the anti-tumor response. In the steady state, resting Treg mainly use oxidative metabolism, however, Treg proliferation is dependent on an oscillatory switch of glycolysis [39]. The anergic state of Treg cells depends on the elevated activity of the mammalian target of rapamycin (mTOR) pathway, and the inhibition of mTOR with rapamycin makes Treg highly proliferative. Activated Treg express high Glut1 for glucose uptake. However, while activated conventional T cells use glucose for aerobic glycolysis, in Treg, a substantial proportion of glucose is converted into pyruvate for mitochondrial oxidization [40]. Proliferating Treg also exhibit increased FAO and enhanced expression of genes involved in FAO during proliferation. Overall, evidence suggests that, compared to conventional T cell subsets, Treg show a selective dependency on oxidative metabolism during proliferation, while being less dependent on glycolysis. The generation of pyruvate for mitochondrial oxidation is required for metabolic fitness and indeed the depletion of glucose is detrimental for Treg proliferation. Interestingly, individuals that carry a loss-of-function mutation in the glucokinase gene display a reduced pool of circulating Treg [41]. Glucose plays also an essential role in Treg’s suppressive function and stability. Under hypoxic conditions, HIF-1a prevents glucose-derived pyruvate from mitochondrial oxidation and promotes glycolysis which is associated with a reduced suppressive function [42]. Treg stability is tightly linked to the expression of FOXP3 which is modulated by the glycolytic activity. Enolase-1 is a glycolytic enzyme that, under glycolytic circumstances, is forced to the cytoplasm instead of binding FOXP3 in the nucleus, preventing it from suppressing FOXP3 expression in human Treg. Glycolysis also promotes the activity of the transcription repressor enhancer of zeste homolog 2 (EZH2), which promotes FOXP3 expression [43]. Thus, targeting glucose metabolism to promote long lasting immunity in conventional T cells can also affect Treg proliferation, suppressive function, and stability, increasing the value of this approach in improving the efficacy of cancer immunotherapy.

## 5. Manipulation of Glucose Metabolism

The targeting of metabolic processes, in particular glucose metabolism, holds a promise as an anticancer strategy, due to the critical role that metabolic pathways play in regulating both T cell function and cancer cell growth. A variety of approaches are under study, thanks to the availability of a broad arsenal of molecules and drugs. The involvement of metabolic circuits in every physiological process requires an extra level of control over side-effects and off-target effects of metabolic treatments. In this scenario, the identification of biomarkers of response is an active field, allowing to personalize the therapeutic approach and avoid detrimental responses.

### 5.1. Pharmacological Manipulation of Glucose Metabolism 

Over the years, substantial efforts have been made to identify and test molecules that interfere with glucose metabolism, mainly inspired by the therapeutic potential of targeting glycolytic cancer cell growth and proliferation. 2-Deoxy-D-glucose (2DG) is a synthetic glucose analog in which the C-2-hydroxyl group is replaced by hydrogen, and it is probably the best characterized and extensively investigated inhibitor of glucose metabolism since the early 1950s [44]. 2DG is transferred in the cytoplasm by the Gluts and therefore acts as a competitive inhibitor of glucose. After entering the cell, 2DG is phosphorylated by hexokinase and converted into 2-Deoxy-D-glucose-6-phosphate (2DG-6-P), which cannot be further metabolized and, accumulating non-competitively, inhibits hexokinase and competitively inhibits phosphoglucoisomerase [45]. By interfering with the first critical steps of glucose metabolism, both glycolysis and OXPHOS may be partially disrupted. 2DG was tested in several clinical trials alone or in combination [https://clinicaltrials.gov/ct2/results?cond=2DG&term=&cntry=&state=&city=&dist=].

Despite relatively mild side effects, the use of 2DG alone was not proven effective and its potential application in combined therapies is still under evaluation. By blocking glycolytic enzymes, 2DG has an effect on basically all cells in the body. More recently, novel molecules that act as selective inhibitors of glucose transporters have been generated and tested. To block a single glucose transporter increases selectivity on specific cellular targets. In this section, we provide a summary of the latest molecules that have been synthetized, focusing on new generation molecules that specifically target Glut1. Three small molecules have gained interest in recent years (Table 1). 

STF-31 is shown to act as a selective Glut1 inhibitor and inhibits the cell growth of the kidney and other types of cancer cells that lack the von Hippel–Lindau (VHL) tumor suppressor protein [46]. The inactivation of VHL enhances the expression of the hypoxia-inducible transcription factor (HIF), which in turn stimulates the transcription of glycolytic genes, including the GLUT1 gene. VHL-deficient cancer cells are dependent on Glut1 and aerobic glycolysis for ATP production. STF-31 binds directly to Glut1 and interferes with glucose uptake, causing necrosis in VHL-deficient cancer cells. Interestingly, normal kidney cells are unaffected, as they do not strictly depend on glycolysis and can alternatively use Glut2 for glucose uptake. STF-31 inhibits tumor growth without significant toxicities and side effects in mouse models. In the immune system, STF-31 was shown to reduce glucose uptake and glycolytic rate in human CD4+ T cells [47]. 

WZB117 is a small molecule acting as a Glut1 inhibitor by reversibly binding the exofacial sugar-binding site of Glut1 and competing with glucose for transport into the cell [48]. WZB117 was shown to induce cell death in lung and breast cancer cells in vitro and in animal models, without showing significant toxicity to normal cells [49]. In the immune system, we showed that WZB117 reduces T cell proliferation by 90% at a concentration of 3 mM and interferes with the differentiation of activated naïve T cells into memory T cells. Both STF-31 and WZB117 efficiently block Glut1 and glucose transport in cancer cells and T cells. However their effect is observed at a micromolar range and the selectivity for Glut1 is still controversial [50], with significant limitations for their translation into clinical trials. So far, BAY-876 has proven the newest and most promising molecule [51], with an IC50 of 2 nM, and highly selective for Glut1 (with a selectivity factor of >100 against Glut2, Glut3, and Glut4). Moreover, it is orally bioavailable, soluble, and displays a good metabolic stability in vitro. As for the two other compounds, BAY876 has shown a potent anti-cancer activity in pre-clinical models [52], but data on its effect on the immune system and more specifically on T cells are not available yet.

### 5.2. Side Effects Related to Manipulation of Glucose Metabolism

Selective Glut1 inhibitors have been proposed as potential treatment for diseases like cancer and autoimmunity. However, preliminary data are not available with respect to potential off-target and side effects, since Glut1 is expressed by a broad range of cells in the body. Gluts expression is also highly redundant and potential targets can be identified as cells that express only Glut1 as well as cells in which Glut1 is highly related to a specific function. Red blood cells express only Glut1, whose function appears more related to the transport of ascorbic acid than glucose [53]. Treatment of mice with STF31 at a dose that inhibits cancer cell growth does not affect red blood cell numbers [54]. Human (but not mouse) insulin-producing beta-cells express Glut1, which is involved in glucose sensitivity and insulin secretion. Treatment of human beta-cells with STF31 results in impaired insulin secretion [55]. The blood brain barrier is probably the best characterized tissue in which Glut1 plays a non-redundant role for glucose transport from blood to the central nervous system. Even though none of the Glut1 inhibitors described in this section has been tested for a potential effect on the BBB, a human genetic Glut1 deficiency syndrome which results in reduced glucose transport has been described and associated with mild to severe neurological symptoms, especially in children and young patients [55,56]. These and potential other side effect need to be carefully investigated for the clinical translation of this approach, but it is likely that the use of a pharmacological Glut1 blockade approach can be applied only for a time-limited therapeutic window and not for a chronic therapy.

### 5.3. Long Term Glucose Restriction through Ketogenic Diets

**Figure 1:**
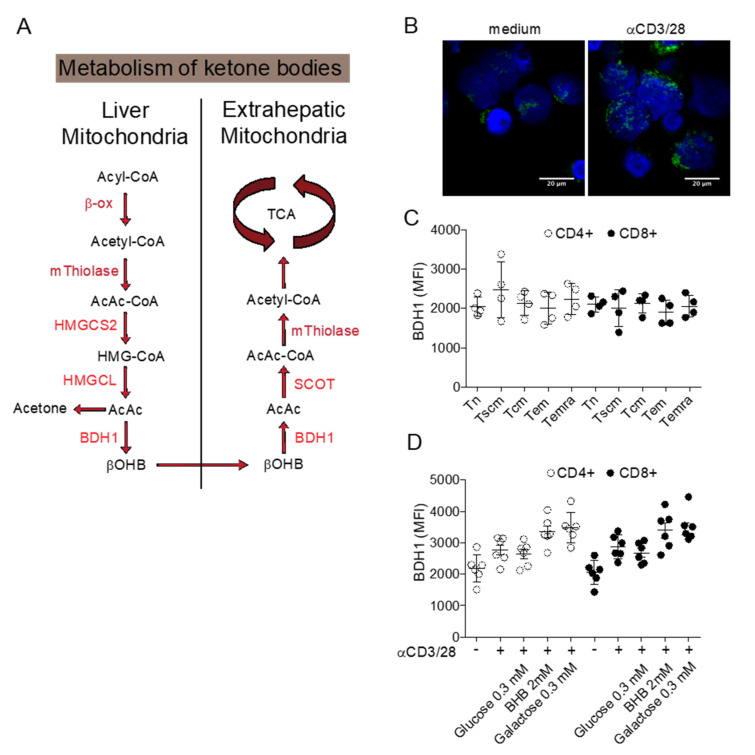
Metabolism of ketone bodies. (**A**) Schematic representation of ketone bodies synthesis in the liver and utilization as metabolic substrate in extra-hepatic tissues. (**B**) Confocal image of BDH1 positive mitochondria in resting and activated T cells. (**C**) Expression of BDH1 in CD4+ and CD8+ T cell subsets. (**D**) Changes in BDH1 expression in CD4+ and CD8+ T cells cultured in vitro in different conditions. BDH1: D-beta-hydroxybutyrate dehydrogenase; SCOT: Succinyl-CoA: 3-ketoacid-coenzyme A transferase; HMGCL: hydroxymethyl-3-methylglutaryl-CoA lyase; HMGCS2: 3-hydroxy-3-methylglutaryl-CoA synthase 2; TCA: tricarboxylic acid cycle.

While the pharmacological inhibition of Glut1 can find therapeutic application as short-term treatment, a maintenance therapy to restrict glucose availability is more difficult to achieve. Diets restricted in carbohydrates are recognized as a sound long-term therapeutic strategy to reduce the availability of glucose in the body and possibly targeting the Warburg effect in cancer cells [57]. Ketogenic diets (KD) are poor in carbohydrates (usually less than 50 g/day) and more abundant in lipids and proteins to preserve an appropriate intake of calories. Under this condition, the body reacts by producing ketone bodies from lipids, as occurs during starvation. Ketone bodies (including acetoacetate, acetone, and β-hydroxybutyrate) are metabolized from acetyl-CoA, which is produced by fatty acid oxidation in the liver and transported to extrahepatic tissues as energy supply (Figure 1A). Acyl chains are transported across the mitochondrial membranes and undergo β-oxidation [58]. The mitochondrial isoform of 3-hydroxymethylglutaryl-CoA synthase (HMGCS2) catalyzes the condensation of acetoacetyl-CoA (AcAc-CoA) and acetyl-CoA to generate HMG-CoA. HMG-CoA lyase (HMGCL) cleaves HMG-CoA and generates acetyl-CoA and acetoacetate (AcAc). AcAc is subsequently reduced to β-hydroxybutyrate (D-βOHB) by phosphatidylcholine-dependent mitochondrial βOHB. A proportion of AcAc spontaneously decarboxylate to acetone. Ketogenesis occurs primarily in the mitochondrial matrix of hepatocytes at a rate proportional to the rate of FAO. AcAc and βOHB exit from cells and are transported to extra-hepatic tissues, where they are catabolized in mitochondria to acetyl-CoA, which is available to the TCA cycle for terminal oxidation. Ketone bodies are used as an alternative energetic fuel in the heart, skeletal muscle, and brain. Extrahepatic mitochondrial BDH1 catalyzes the first reaction of βOHB oxidation, converting βOHB back to AcAc. 

The rationale to associate KD to standard cancer treatments is to target the Warburg effect as cancer cells predominantly utilize glycolysis instead of oxidative phosphorylation to produce ATP. Furthermore, some cancers have an impaired capacity to metabolize ketone bodies, due to mitochondrial dysfunction and the down-regulation of enzymes for ketone bodies’ metabolism [59]. Cancer cells display mitochondria abnormalities, appear swollen, and exhibit cristolysis. The structural abnormality is consistent with functional deficiencies in the respiratory capacity. These observations prompted researchers to test ketogenic diets as adjuvant to chemotherapy, showing beneficial effects in some tumors including glioblastoma, neuroblastoma, prostate, colon, pancreatic and lung cancer but not in others such as astrocytoma and medullo-blastoma, kidney cancer, and melanoma [60]. Overall, these results indicate that KD can contribute to suppress tumor growth and to sensitize tumors to chemotherapy in some cancers. 

In the context of cancer immunotherapy, a ketogenic diet regimen can represent an advantage if T cells are able to efficiently utilize ketone bodies. We conducted preliminary studies to determine whether T cells can metabolize ketone bodies. Resting human T cells express the enzyme BDH1 in T cell mitochondria (Figure 1B) and T cell activation causes the increase of the mitochondrial T cell mass further enhancing the expression of BDH1. In resting CD4+ and CD8+ T cells, BDH1 is expressed at similar levels in naïve, stem cell memory, central memory, effector memory, and terminally differentiated effector memory T cell subsets. BDH1 expression can be increased by antigenic activation, by the addition of the BDH1 substrate βOHB, and by blocking glycolysis with galactose. Overall, these preliminary results indicate that T cells display the bio-chemical machinery to metabolize ketone bodies and further investigations are required to better characterize the positive and negative effects of ketogenic diets in T cell metabolism and its possible application in cancer immunotherapy.

## 6. Relevance of Immunometabolism in Cancer Immunotherapy

The condition of the nutrient deprivation characteristics of tumor tissues severely impact the effectiveness of the antitumor immune response [61,62,63]. Metabolic reprogramming, a hallmark of cancer progression, is tightly linked to immune cell function, suggesting therapeutic modulation of metabolic pathways as a strategy to enhance antitumor immune response. This becomes particularly important in the design of immune-based anticancer strategies, aimed at enhancing the protective immune responses that can eliminate established tumors. Several immunotherapy approaches have been designed and tested and include therapies based on cytokine administration, antibodies targeting specific receptors, adoptive cell transfers, vaccinations with cancer antigens, genetically engineered chimeric antigen receptor (CAR) T cells, and immune checkpoint blockade (ICB). Encouraging results have been variably observed, depending on the tumor type and stage of disease. 

### 6.1. Manipulation of Metabolism in Immunotherapy

Among other immune-based approaches, inhibitors of immune checkpoints, i.e., antibodies that disrupt the receptor/ligand pairs inhibiting effector T cells (e.g., Programmed Death-1 [PD-1] and Programmed Death-Ligand 1 [PD-L1]), are FDA-approved as treatment options in patients with many types of advanced cancers [64,65]. Despite notable clinical benefit, typically <50% of patients receiving checkpoint inhibitors experience objective, durable responses. Detailed analyses of the immune landscape of cancer tissues have shed light on the variety of factors that can interfere with ICB and predict response to the treatment [66]. Type, density, and location of immune cells hold a key position as clinical variables of response [67], together with those environmental factors that can affect immune activation, including metabolic cues. Preclinical evidence points to metabolic perturbations during ICB treatments, whereby tumor-reactive cytotoxic T lymphocytes display increased mitochondrial mass and more reactive oxygen species (ROS) [68]. Consistently, metabolic variables have shown clinical association with response to checkpoint inhibitors in cancer patients [60,61,62,63,64,65,69,70,71,72,73,74], suggesting that markers of T cell metabolism may serve as monitoring biomarkers in the PD-1 blockade therapy.

**Table 1:** Basic characteristics of small molecules that act as Glut1 inhibitors.

Compound	Structure	MW	IC50 (μM)	Selectivity for Glut1	References
STF-31	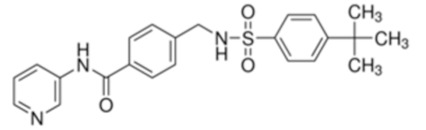	423.53	1	Low	[46,47,50]
WZB-117	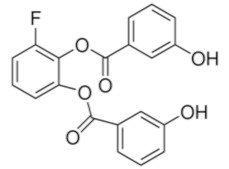	368.31	0.5	Low	[48,49,71]
BAY 876	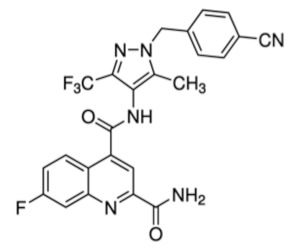	496.42	0.002	High	[51,52]

Three small molecules targeting glucose metabolism by inhibiting Glut1 transporters are listed. Data were compiled from listed references. STF-31: 4-[[[[4-(1, 1-Dimethylethyl) phenyl] sulfonyl] amino] methyl]-N-3-pyridinyl-benzamide. WZB-117: 3-Fluoro-1, 2-phenylene bis (3-hydroxybenzoate), 3-Hydroxy-benzoic acid 1, 1′-(3-fluoro-1, 2-phenylene) ester. BAY-876:N4-[1-[(4-Cyanophenyl) methyl]-5-methyl-3-(trifluoromethyl)-1H-pyrazol-4-yl]-7-fluoro-2, 4-quinolinedicarboxamide. MW: molecular weight; IC50: half maximal inhibitory concentration.

A logical consequence of this immune-metabolic link is the possibility to target metabolism to improve immune-based strategies. One interesting and attracting approach is the use of glucose-limiting lifestyle changes though specific dietary regimens or anti-diabetic drugs that can be co-administered with immunotherapy. The rationale underlying this approach is to target the cancer-cell sustaining “Warburg effect” [5]. Thus, low carb diets and pharmacologic interventions that reduce glucose available to cancer cells levels may slow cancer cell replication rate and render cancer cells more susceptible to immune-mediated killing, thereby improving the effectiveness of immunotherapy. On the other hand, limiting glucose availability can have unintended negative consequences for protective immunity. Indeed effector CD8+ T cells also rely on aerobic glycolysis for clonal expansion and to acquire effector functions, including cytolytic activity and cytokine secretion [3]. Prior studies report that dysregulated CD8+ T cell metabolism impairs T cell effector functions and promotes tumor progression within the tumor microenvironment [63]. For instance, in subjects with clear cell renal cell carcinoma, metabolic defects of tumor-infiltrating CD8+ T cells, including a reduction of proliferative capacity, impaired glycolysis and increased production of reactive oxygen species have been associated with a low availability of glucose. In this context, further limitations of intra-tumoral glucose could severely impair T cell metabolism and function. However, it has been shown that ICB may selectively protect T cells from reduced glucose availability within the tumor microenvironment [63]. In the report, ICB administration with either anti-CTLA-4, anti-PD-1, or anti-PD-L1 restore the glycolytic capacity and production of IFN-gamma in CD8+ tumor-infiltrating T cells. Interestingly, anti-PD-L1 administration inhibited glycolysis in tumor cells. These data suggest that ICB is a particularly attractive type of immunotherapy in which glucose-limiting interventions may simultaneously be impaired tumor cell viability and boost T cell effector function. Limited preclinical and clinical data were generated to determine if glucose-limiting lifestyle interventions can affect other immunotherapy platforms, such as adoptive cell therapies, or CAR T cells.

### 6.2. A Model to Integrate Metabolic Interventions and Cancer Immunotherapy

**Figure 2:**
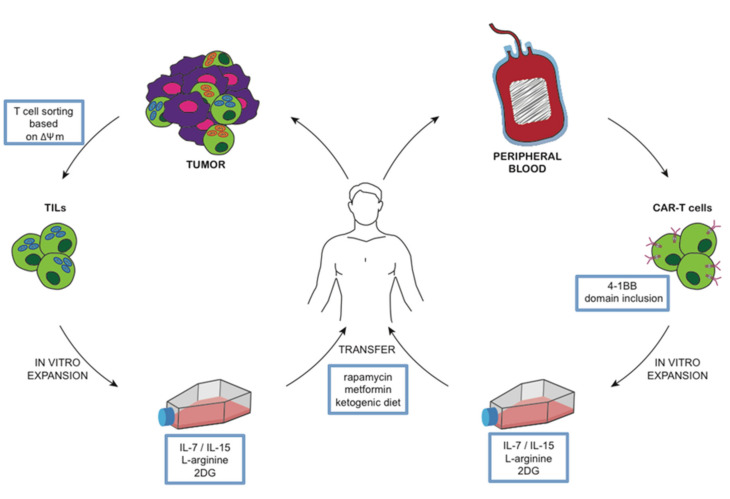
Model of metabolic manipulation of T cells. T cells obtained from patients and expanded in vitro can be manipulated in different metabolic pathways. In this way, it is possible to generate cancer specific T cells (TIL or CAR T cells) that, once infused back into the donor, are expected to present with improved performances in terms of longevity, persistence, and anticancer activity.

Current procedures for adoptive T cell therapy involve three stages that are amenable to metabolic manipulation (Figure 2). The first stage is the selection of primary cells from patients. This mainly applies to ex-vivo isolation of tumor infiltrating lymphocytes (TIL) for in vitro expansion. A promising strategy is to isolate TIL according to their mitochondrial membrane potential (ΔΨm) [75], suggesting a novel approach to isolate therapeutic T cells based on metabolic fitness. The selection of T cells is based on the uptake of the lipophilic cation dye tetrametylrhodamine methyl ester (TMRM), allowing to discriminate between CD8+ T cells with a low or high ΔΨm. T cells sorted according to a low ΔΨm showed a better metabolic fitness during in vitro expansion and an improved capacity of the long-term persistence and eradication of established tumors once transferred in vivo in animal models. This method allows ex vivo isolation of metabolically improved T cells, is potentially applicable to both CD4+ and CD8+ T cells, and can be used to avoid the presence of unfit T cells in the initial preparation of T cells. Most protocols for the generation of therapeutic T cells involves protocols for extensive in vitro expansion in order to obtain a sufficient number of cells for an effective anti-tumor response upon in vivo transfer. An enhanced glycolytic metabolism is associated with increased expansion, early proliferation, and production of effector cytokines, such as IFN-γ. On the other hand, an oxidative metabolism promotes the generation of memory clones, with a less potent effector function but with a superior long-term survival and anticancer effectiveness in vivo. Several approaches have been tested to modify the metabolic signature of T cells. The adoptive transfer of T cells expanded in vitro in the presence of 2DG to limit glycolysis, showed and enhanced anti-tumor activity in a mouse model of melanoma [24]. In mouse models of melanoma, pharmacologic inhibition of the serine/threonine kinase Akt achieved by using Akt inhibitor VIII (PubChem Compound Identification: 10196499) during in vitro expansion of TIL has led to the generation of a long-lived and memory-like T cell pool with potent anti-tumor activity [76]. Metabolic manipulation was also tested in CAR T cells to improve longevity and to induce a memory-like phenotype. The 4-1BB co-receptor signaling domain was included into the CAR architecture, inducing mitochondrial biogenesis and oxidative metabolism that associated with a central-memory phenotype and improved longevity and persistence [77]. In contrast, the inclusion of the CD28 signaling domain resulted in highly glycolytic T cells with improved effector function but reduced persistence. 

Cytokines and nutrients can be added to T cell culture media to manipulate metabolism. IL-7 and IL-15 were used to generate T cells with a stem cell memory phenotype during polyclonal expansion from naïve precursors [78]. Naïve T cells that recognize autoantigens are included in the T cell repertoire from birth [79]. We showed that priming of naïve T cells with autoantigens in the presence of IL-7 resulted in an expanded population of antigen-specific T cells with a Tscm phenotype and function [80]. While these T cells require glucose, they produce little lactate and preferentially oxidize pyruvate in the mitochondria in order to generate Tscm. Moreover, IL-7 is able to compete with IL-2 for engagement of the common γ chain [81], possibly reducing the differentiation of effector T cells in response to autocrine IL-2 production. Naïve T cells undergo a reduction in intracellular L-arginine content upon activation. The supplement of L-arginine in culture conditions reduced the use of glycolysis and promoted mitochondrial oxidation associated with a central-memory phenotype and improved anti-tumor activity in mouse models [82]. 

Once transferred in patients, metabolic manipulation becomes more difficult to achieve. We mentioned selective Glut1 inhibitors as potential metabolic modulators and the possibility to adopt a ketogenic diet. In this section, we will focus on molecules that are commonly used in clinical practice and can exert an important metabolic effect and are therefore interesting as potential metabolic adjuvant in patients undergoing to cancer immunotherapy. The immunosuppressive drug rapamycin is an mTOR-signaling inhibitor able to interfere with the gene expression program involved also in cancer metabolic reprogramming. Everolimus, a rapamycin analog that specifically inhibits mTORC1, has been tested in clinical trials for metastatic renal cell carcinoma [83]. Rapamycin administration in vivo was shown to interfere with the generation of memory T cell responses [84]. The treatment of mice with rapamycin following acute lymphocytic choriomeningitis virus infection was shown to enhance the quantity and the memory like-phenotype of CD8 T cells. Similar effects were observed also in non-human primates following vaccination with modified vaccinia virus Ankara. 

Metformin is a commonly prescribed drug for type II diabetes and is currently being tested in clinical trials for its potential antineoplastic activity [85]. Metformin can also ameliorate the immune response to tumors. Metformin administration was shown to induce regression in highly immunogenic tumor cells (leukemia and renal carcinoma cell) in mice. This was mediated both by a direct effect of the drug on CD8+ TIL in which metformin prevents apoptosis and exhaustion and increases effector-memory T cells population within the tumor tissue [85]. In conclusion, of the multiple processes that are required to perform an adoptive cancer immunotherapy, most of these processes are amenable for metabolic intervention to promote a memory T cell response over an effector T cell response in order to improve the effectiveness of T cells against cancer. 

Notably, some of the metabolic modulators described in this review can alleviate cancer cachexia, a frequent and debilitating condition associated with several cancer subtypes. Cancer cachexia is a wasting syndrome characterized by weight loss, anorexia, asthenia, and anemia. The pathogenesis of this syndrome has not been fully elucidated but involves several metabolic and immune-mediated mechanisms, recently reviewed in [86]. In animal models, a ketogenic diet was proven effective in mitigating cancer cachexia, acting both on nutrient availability and by reducing systemic inflammation [87]. A ketogenic diet has also been administered to cachectic cancer patients showing a reduction in gluconeogenesis and circulating lactate, but there was no effect on protein turnover [88]. More recently, a ketogenic diet was shown to improve the metabolic profile of pancreatic cancer patients [89]. Metformin was successfully used to reduce wasting effects in muscle protein metabolism tumor-bearing rats [90]. Similarly, rapamycin was shown to reduce autophagy and rescue muscle mass in tumor bearing mice [91]. Although not conclusive, this data indicates that metabolic manipulation in cancer patients can exert positive effects beyond the specific aim of reducing cancer cell growth and improve anti-cancer T cell responses. 

## 7. Conclusions

Metabolic alterations have long been recognized as a hallmark of cancer, nonetheless, only recently have we started to appreciate metabolic reprogramming in immune cells during their activation. Our growing understanding of the complex metabolic interplay that regulates immune function in cancer tissues is projected to reshape anticancer strategies, integrating pharmacological control of metabolic cues with immune targeting. The careful identification of biomarkers of response to prevent side-effects is ongoing.
